# Sunroot snack bar: Optimization, characterization, consumer perception, and storage stability assessment

**DOI:** 10.1002/fsn3.2412

**Published:** 2021-06-29

**Authors:** Tarek Gamal Abedelmaksoud, Sayed Saad Smuda, Ammar B. Altemimi, Reda Mahgoub Mohamed, Anubhav Pratap‐Singh, Marwa Rashad Ali

**Affiliations:** ^1^ Food Science Department Faculty of Agriculture Cairo University Giza Egypt; ^2^ Department of Food science College of Agriculture University of Basrah Basrah Iraq; ^3^ Food, Nutrition & Health Program Faculty of Land and Food Systems The University of British Columbia Vancouver BC Canada

**Keywords:** oats, snack bar, storage stability, sunroot, vegetable

## Abstract

This study reports the evolution of phenolics, inulin content, proximate composition, hardness, and sensory characteristics of an inulin‐rich healthy snack bar (The Sunroot Snack Bar) over 90 days of storage in refrigerated and room temperature storage. A response surface methodology (RSM) with a central composite rotatable design was first employed for optimizing the concentrations of sunroot, potato, and oats. The optimum selected concentrations of sunroot, potato, and oat were 53.99, 37.88, and 5 g, respectively, and a quadratic model was found to yield the best fit. Analysis of variance revealed that a higher sunroot content resulted in more firmness of the bar and higher overall acceptability in sensory trials. Sunroot snack bar samples without flavor (control), sunroot snack bar with cheese flavor (S1), and sunroot snack bar with olive flavor (S2) were then tested for sensory, chemical, phytochemicals, and microbial contents among control, S1, and S2 samples over a 90‐day shelf‐life study. Results showed no significant (*p* < .05) changes in these contents on addition of flavor. An increase in microbial load and the appearance of a bitter taste after 30 days of fresh sunroot storage were observed. No microbial growth was observed in all sunroot snack bar samples during storage at 4°C, while some microbial growth was observed at 25°C for 90 days. It was inferred that the high‐quality shelf life of the sunroot bar was 90 days at 4°C, which was shortened to a month if the bars were preserved at 25°C. There was a significant phenolic and inulin content loss at 25°C compared with 4°C in total phenolic component. Based on the results of sensory evaluation, online questionnaire of customer experience, and cost analysis, this study successfully used sunroot tubers for the production of snack bars as a promising new raw material, which was introduced healthily with a suitable price for such product compared with other products in the market.

## Graphical abstract

This study reports the evolution of phenolics, inulin content, proximate composition, hardness, and sensory characteristics of an inulin‐rich healthy snack bar (The Sunroot Snack Bar) over 90 days of storage in refrigerated and room temperature storage. Based on the results of sensory evaluation, customer experience questionnaire, and cost analysis, this study successfully used sunroot tubers for the production of snack bars as a promising new raw material.

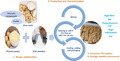

## 1 INTRODUCTION

Consumption of fresh fruit and vegetables is an important part of a healthy diet as their natural antioxidants and dietary fibers help reduce the risk of chronic diseases. As a result, the twenty‐first century has presented a growing consumer demand for healthy foods (Pratap‐Singh & Ramaswamy, 2016). Therefore, attempts are being made to incorporate healthy ingredients, such as dietary fibers, antioxidants, and micronutrients, such as vitamins, healthy sugars, or minerals, to produce food products that alleviate health problems (Pelucchi et al., 2004; Radovanovic et al., 2019; Seeram et al., 2005). Nowadays, numerous fiber sources, including whole plant cell walls, and non‐starch oligo and polysaccharides like inulin and pectin (Ferguson et al., 2001), are used as an additive to provide qualifying properties, such as water holding capacity in food applications (Asha & Pratima, 2009).

Inulin is considered an important functional ingredient (González‐Herrera et al., 2015) due to its low glycemic index (GI) that can reduce the risk of chronic disorders like diabetes, coronary cardiovascular disease, obesity, strokes, and cancers (Barclay et al., 2008). Also, inulin enhances digestive health and prevents constipation. Inulin can reduce blood cholesterol and lipids, and improve absorption of mineral from the colon, due to which it is considered as a prebiotic material (Barclay et al., 2008; Watzl et al., 2005).

Sunroot, also known as Jerusalem artichoke, belongs to the family Asteraceae and genus Helianthus and is a species of sunflower native to Central North America. It contains high amount of inulin, which is considered a prebiotic that could be used for preparing low‐calorie functional food fortified. Sunroot also contains a high percentage of phenolic compounds with high antioxidant potential, such as sesquiterpenes, polyacetylenic derivatives, and coumarins (Yuan et al., 2012). Moreover, Showkat et al. (2019) conducted that the HPLC‐UV analysis of main phenolic acids in Jerusalem artichoke tubers was chlorogenic acid, p‐coumaric acid, caffeic acid, and the dicaeoyl isomers 1,5‐diCQA and 3,5‐diCQA, which were extracted using solvents or microwave method. In general, sunroot comprises 80% water, 15% carbohydrate, and 1% to 2% protein, no fat, and little starch. When stored for some time (typically months), the inulin content gets converted into fructose (it is rich in inulin, 8%–13%), which imparts the characteristic sweet taste (Kays & Nottingham, 2007). Fresh sunroot tuber is very perishable with a shelf life of not more than 30 days at refrigerator, thus requiring immediate processing into value‐added products to ensure sustainability of sunroot farming.

Therefore, the use of sunroot as an ingredient to produce an inulin‐rich functional food product is plausible. Fruit and vegetable snack bars are concentrated products that have outstanding nutritional and energy attributes for all ages, including old people (Parn et al., 2015). In addition, fruit and vegetable snack bars have a long shelf life compared with fresh ones. Due to their popularity among all populations, and especially the elderly people (Parn et al., 2015), fruit and vegetable snack bars may be the ideal food format/vehicle to supply phenolic compounds and dietary fiber derived from fruit and vegetables, required to meet human daily requirements (Sun‐Waterhouse et al., 2010). Various studies used vegetables and fruits to produce snack bars for example banana, sweet potato, and beetroot (Mahendradatta et al., 2020; Maity et al., 2016; Sunyoto et al., 2019). From the previous literature, there is no publication available for using sunroot as a good source of inulin and phenolic compounds to produce snack bars. The objective of this study was to produce a ready‐to‐eat sunroot snack bar rich in inulin and phenolic and to assess its nutritional quality and storage stability during 90 days of storage at 4 and 25°C.

## 2 MATERIALS AND METHODS

### 2.1 Raw material

Sunroot (Helianthus tuberosus) was harvested from the farm of vegetable department, Faculty of Agriculture, Cairo University (Cairo, Egypt). Potato (*Solanum tuberosum*), nuts (Peanuts), eggs, natural cheese flavor, olive flavor, creamy cheese, and oats (converted to powder) were purchased from a local market in Giza, Egypt. Sunroot was sorted, washed, and peeled, and buds were removed. It was then blanched in water at 85°C for 20 min and immediately cooled to room temperature, and later drained to remove excess water until it turned into a paste. All chemicals used in chemical analysis were bought from Sigma‐Aldrich Chemical Co., UK, while chemicals used in microbial analysis were purchased from Oxoid, Hampshire, UK.

### 2.2 Optimization of sun root snack bar

Sunroot paste, potato paste, oat powder, creamy cheese, roasted nuts, and olive or cheese (as natural flavor enhancers) were used to prepare sunroot snack bar. The amount (g) for sunroot paste, potato paste, and oat powder (experimental variables) was optimized by response surface methodology (RSM) using a central composite response surface design generated from Design‐Expert version 7.0.0 (Statease Inc., Trial version). Twenty experiments were carried out with different permutations of selected variable components. Based on the pretrials (Tables S3 and S4), the ranges of values were 30–60 g sunroot paste, 30–50 g potato paste, and 5–15 g oat powder. The experimental strategy is given in Table 1 in the actual form of process variables (sunroot, potato, and oats levels) along with responses (firmness and overall acceptability values). To optimize the impact of unexplained variability in the observed responses because of extraneous variables, experiments were randomized.

**TABLE 1:** Experimental design used and values of response

Run	Sunroot (g)	Potato (g)	Oats (g)	Firmness (N)	Over all acceptance
1	52.37	50.00	5.00	33.99 ± 0.11	7.94 ± 0.09
2	41.19	40.00	10.00	34.68 ± 0.12	7.74 ± 0.12
3	52.37	30.00	5.00	33.41 ± 0.15	8.22 ± 0.10
4	41.19	32.37	10.00	34.65 ± 0.13	7.91 ± 0.02
5	41.19	40.00	10.00	34.89 ± 0.19	7.83 ± 0.06
6	52.37	50.00	15.00	36.07 ± 0.15	6.38 ± 0.07
7	41.19	40.00	10.00	34.87 ± 0.13	7.75 ± 0.11
8	60.00	40.00	10.00	35.19 ± 0.11	7.87 ± 0.02
9	41.19	40.00	18.41	36.08 ± 0.11	6.38 ± 0.04
10	41.19	40.00	10.00	34.89 ± 0.10	7.71 ± 0.11
11	41.19	56.82	10.00	34.99 ± 0.19	6.44 ± 0.09
12	41.19	40.00	3.41	33.27 ± 0.12	8.45 ± 0.07
13	30.00	50.00	15.00	35.52 ± 0.14	6.05 ± 0.08
14	52.37	30.00	15.00	35.43 ± 0.18	7.26 ± 0.02
15	30.00	30.00	5.00	33.08 ± 0.16	7.81 ± 0.19
16	30.00	30.00	15.00	35.47 ± 0.18	6.85 ± 0.16
17	30.00	50.00	5.00	33.47 ± 0.16	7.85 ± 0.09
18	32.66	40.00	10.00	34.53 ± 0.14	7.64 ± 0.11
19	41.19	40.00	10.00	34.9 ± 0.15	7.78 ± 0.13
20	41.19	40.00	10.00	34.91 ± 0.17	7.73 ± 0.10

#### 2.2.1 Sunroot snack bar packaging and storage conditions

All ingredients of respective formula were thoroughly mixed and then spread on aluminum trays into a 2.5 cm thick layer. Trays were then baked in an electric oven (Kiriazy, Italy) at a temperature of 160°C for 1 hr followed by 140°C for 30 min. At the end of the baking procedure, trays were removed from the oven and cooled at room temperature and subsequently cut into slabs of a suitable size (about 2.5 cm × 7 cm). Sunroot snack bar samples (30 g for each one) were packaged in pouches of polypropylene (PP) (thickness of 30 mm, 12 × 10 cm^2^), heat sealed and stored for 90 days either at 4 or 25°C. The determination of both chemical and microbiological profiles in sunroot snack bar samples were conducted after 15, 30, 60 and 90 days.

#### 2.2.2 Methods of analysis

The determination of moisture, protein, lipids, crude fiber, and ash contents was conducted as described in the basic protocol of Horwitz and Latimer, AOAC, (Horwitz & Latimer, 2010).

The content of carbohydrates was calculated with some modifications by the phenol‐sulfuric acid technique as defined in the basic Dubois protocol (Dubois et al., 1956). The extracted sample (0.6 ml) was mixed with the phenol solution (0.3 ml) and vortexed for 5 s. Then, 1.5 ml of concentrated sulfuric acid was added to the liquid surface (either directly or slowly down the tube side). Next, all tubes were closed, vortexed for 5 s, and incubated for 30 min at room temperature (20–22°C) and the absorbance was measured at 490 nm. Distilled water was used as a blank.

Inulin content determination was conducted by spectrophotometric method according to the protocol described by Rane et al. (2018). The results of inulin content were expressed as % w/w.

Microbial count (i.e., total plate counts and yeast and mold count) was carried out according to Andrews (1992) with some modification as in Abedelmaksoud et al. (2019).

Total phenolic compounds were determined by the Folin–Ciocalteu system according to Abedelmaksoud et al. (2019) with minor changes reported by Wiktor et al. (2019). In general, 1 ml of each sample (pre‐evaporation extract) was applied to the test tube and 1 ml of Folin–Ciocalteu reagent was added. 1 ml of sodium carbonate (7.5%) was added after 3 min. The mixture remained in the dark for 1 hr, and the absorbance was measured at 740 nm. The total content of phenolics was determined using a standard Gallic acid curve and expressed as a sample of mg Gallic acid/100 g.

HPLC analysis of phenolic compounds was conducted as follows: The extract of each sample was analyzed using an Agilent 1260 series HPLC system (Agilent technologies Inc.). The separation was passed using C18 column (100 mm × 4.6 mm i.d., 5 μm). The mobile phase contained of (A) water 0.2% H3PO4, (B) methanol, and (C) acetonitrile at a flow rate 0.6 ml/min. Gradient elute was as per the next scheme: 0–11 min (96% A, 2% B); 11–13 min (50% A, 25% B); 13–17 min (40% A, 30% B); 17–20.5 min (50% B, 50% C), and 20.5–30 min (96% A, 2% B). Detection wavelength was set at 284 nm. The injection size was 20 μl, and the column temperature was maintained at 30°C. Compounds were known by comparing their retention time with those from authentic standards. Calibration curves were utilized to evaluate the compound amounts.

The antioxidant activity of each sample was measured according to the Li et al. (2010) method of DPPH (2, 2‐diphenyl‐1‐picrylhydrazyl) and estimated according to Equation 1:

(1) $$\text{Antioxidant activity}\;\%=\left[\left({\text{abs}}_{\text{blank}}‐{\text{abs}}_{\text{sample}}\right)/{\text{abs}}_{\text{blank}}\right]*100$$

where abs_blank_ and abs_sample_ refer to the absorbance of blank and sample, respectively.

Using a texture analyzer (TAHdi; Secure Micro Systems) fitted with a 5 kg load cell and Warner–Bratzler shear blade works at a test speed of 0.5 mm/s, the firmness of the bars was determined in terms of shear force. The pre‐ and postspeeds, respectively, were set at 1 and 5 mm/s.

Sensory quality characteristics including color, odor, taste, texture, and overall acceptability (OAA) of the sunroot snack bar were evaluated on a standardized nine‐point hedonic scale to determine the most suitable processing parameters according to Larmond (1977) for making snack bars with good eating quality characteristics by a semi‐trained panel consisting of ten members including scientists. According to the following summary, panelists were asked to score the samples for their quality attributes: 0–2 = severe dislike, 3–4 = mild dislike, 5 = fair, 6–8 = moderate like, and 9 = excellent. Samples were drawn randomly from each experimental block, coded and served in a sensory laboratory to the panelists. After tasting each sample, potable water was given to each panel member for rinsing the palate.

#### 2.2.3 Consumer perception

The online questionnaire by google form (a free tool designated for online surveys) was conducted, which the link was sent to participants by email and social media (i.e., WhatsApp and Facebook). A brief explanation was introduced to clarify the present study (typing, calling by cell number, or small voice during the contact by participants) and also contact us for any questions by e‐mail and mobile. The present study does not represent a specific sector of the market or the entire market population, but explores the relationship of consumers with fruit and vegetable bars, including sunroot bar in general, and their motivation to buy such kinds of bar. The respondents to the google form questionnaire were 274:53.6% female and 46.4% male, the age varied from 15 to 69 years. The educational level was less than High School (1.5%), High School/GED (4.9%), Some college (26.6%), 4‐year college degree (46.1%), Master degree (7.9%), and Doctoral degree (13.1%). Number of children in the family were categorized as follows; 1–3 children (69.6%), 4–6 children (29.5%), and more than 6 (4.5%). To have an insight into whether or not consumers perceived fruit or vegetable bar (sunroot bar) as a source of nutritional ingredients, respondents were asked to express their level of agreement as described by Torres et al. (2020). The survey was classified into three parts as follows:

Part 1 was concentrated on the demographic data such as age, gender, education level, and number of children in the family during the first three questions. After that, the rest of the questions were concentrated on the objective, which was to see the consumer perception degree about fruits and vegetables bars in general and sunroot bar in particularly. In this part, a picture of the product, small voice, and call were introduced to identify and know what sunroot product is. The questions for the respondents were as follows:

- Did they know sunroot plant and its nutritional value?
- Which form more comfortable for you as a snack between diets or during work?
- Do you know what the fruit or vegetable bar?
- Did they know what the sunroot plant and its nutritional value?
- How many total servings you eat of fruit or vegetable bar per day?
- Did you hear about functional food?
- Could you purchase functional food instead of commercial once?
- Which consumer groups are the most consuming low calories food?
- If sunroot bar was produced as new products, will you buy it if you know its consider a good source for minerals and phytochemical compounds which help in lower the risk of different diseases such as intestinal problems, heart illness, cancer and don't cause tooth decay because most of their carbohydrate are fiber.
- How well does sunroot bar meet your needs?
- If you could change just one thing about our product, what would it be?
- How would you rate the value for money of the product (one bar 100 g for 3.05–3.13 LE)?
- Shelf life of this product is 3 months?
- Compared with other types of fruit or vegetable bar you tried, is our product nutritional value……?

**FIGURE 1:**
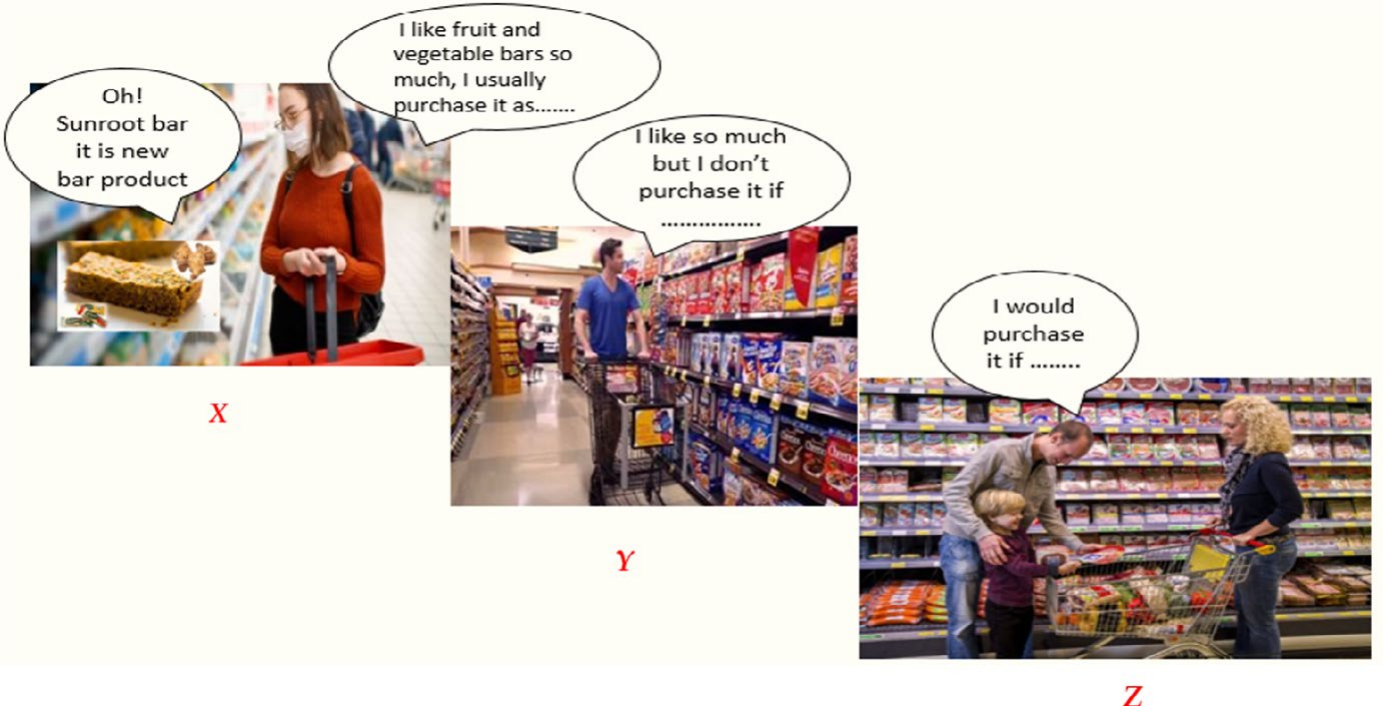
Shows the three (*X, Y, Z*) stimuli dialogue used in the completion task

Part 2: The stimuli dialogue was used in the completion task. Three stimulus were used as follows (Figure 1): First stimulus "Oh! Sunroot bar it is new bar product‐ I like fruit and vegetable bars so much, I usually purchase it as……."; Second stimulus "I like it so much but I will not purchase it if……………."; Third stimulus "I would purchase it if ……..." (Li et al., 2010).

The obtained data were analyzed as described by (Torres et al., 2020). The triangulation technique was used to select either words or phrases written by participants, which three experienced researchers have analyzed the responses and made them in groups according to the personal interpretation. These groups were combined to define the categories, which each group was analyzed according to the number of times mentioned. The analysis was concerned with the categories that represent more than 5% of the answers. This was done in order to avoid the loss of a large amount of information (Guerrero et al., 2010).

#### 2.2.4 Statistical analysis

Response data were fitted to the second order polynomial equation, which described the effect of the independent variables on the response as well as the combined effect of them on the response Y and determined the interrelationship among the test variables. The generalized second‐order polynomial model was used in the response surface analysis, which is described by (Equation 2):

(2) $$Y=a_{o}+a_{1}\chi_{1}+a_{2}\chi_{2}+a_{3}\chi_{3}+a_{12}\chi_{1}\chi_{2}+a_{13}\chi_{1}\chi_{3}+a_{23}\chi_{2}\chi_{3}+a_{11}\chi_{1}^{2}+a_{22}\chi_{2}^{2}+a_{33}\chi_{3}^{2}$$

where *Y* is the expected response of firmness and OAA, respectively, *a*_o_ the estimated regression coefficient of the fitted response at the central point of the model, *a*_1_, *a*_2_, *a*_3_ the coefficient of regression for linear effect expressions, *a*_11_, *a*_22_, *a*_33_ the quadratic effects, *a*_12_, *a*_13_, *a*_23_ the effects of interaction, and *χ*_1_, *χ*_2_, *χ*_3_ are the independent variables sunroot, potato and oat concentrations.

For the optimization of snack bar components (Sunroot, potato, oats) for firmness and OAA values, a desirability function was used. The primary objective of optimization in this study was to maximize overall acceptance (OAA) and firmness in range (response), so Equation 3 describes the desirability function:

(3) $$d\left(y\right)=\left\{\begin{array}{cc} 1 & y\leq L\\ \frac{\left(y‐L\right)}{\left(U‐L\right)} & L<y<U\\ 0, & y\geq U \end{array}\right.$$

where *L* and *U* are, respectively, the lower and upper limit values of the response (*y*). A trial version of Design‐Expert version 7.0.0 (Statease Inc., Trial version) was used to maximize the polynomials via the desirability function process.

A statistical analysis of the findings was conducted by Duncan's multiple range analyses, analysis of variance (ANOVA) and the least significant difference (LSD, 95%) test using XLSTAT software (Addinsoft).

## 3 RESULTS

### 3.1 Ingredients optimization for production of Sunroot snack bars

Sunroot snack bars values: sunroot paste range of 30–60 g, potato paste range of 30–50 g, and oat powder range of 5–15 g were selected (Table 1) for RSM to evaluate the effect of their concentrations on firmness and overall acceptability (OAA) values as well as to optimize process parameters. The ranges of values were selected based on pretrails, and sensory evaluation was done for all pretrails (Tables S3 and S4). The sensory evaluation either less or more than the selected range lead to statistically low sensory evaluation compared with the selected range.

The bar firmness varied between 33.08 and 35.52N (Table 1). For various combinations of variables, the overall acceptability (OAA) values ranged from 6.05 to 8.55. Table 2 shows the effect of sunroot, potatoes, and oats concentration on the Firmness and OAA in the snack bar at 95% confidence interval using ANOVA analysis.

**TABLE 2:** Regression coefficients of the fitted second‐order polynomials representing the relationship between the responses and variables

Factor	Firmness (N)				OAA			
	Coefficients estimate	Sum of squares	*F*‐value	*p*‐Value Prob > *F*	Coefficients estimate	Sum of squares	*F*‐value	*p*‐Value Prob > *F*
Intercept	34.91	14.36	143.06	<0.0001*	7.82	8.73	268.44	<0.0001*
*χ*_1_ (Sunroot)	0.25	0.34	30.06	0.0003*	0.15	0.13	35.07	0.0001*
*χ*_2_ (Potato)	0.25	0.51	45.92	<0.0001*	−0.27	0.63	174.80	<0.0001*
*χ*_3_ (oats)	1.03	10.89	977.08	<0.0001*	−0.65	4.30	1,188.48	<0.0001*
*χ*_1_ *χ*_2_	0.13	0.076	6.82	0.0260*	−0.067	0.020	5.53	0.0405*
*χ*_1_ *χ*_3_	−0.057	0.014	1.30	0.2815	0.040	7.200E−003	1.99	0.1885
*χ*_2_ *χ*_3_	−0.035	9.800E−003	0.88	0.3706	−0.18	0.26	71.71	<0.0001*
$\chi_{1}^{2}$	−0.035	2.990E−003	0.27	0.6158	−0.11	0.028	7.71	0.0196*
$\chi_{2}^{2}$	−0.086	0.060	5.36	0.0432*	−0.31	0.79	219.17	<0.0001*
$\chi_{3}^{2}$	−0.17	0.32	28.42	0.0003*	−0.11	0.12	32.53	0.0002*
Residual		0.11				0.036		
Lack of fit		0.073	1.91	0.2476		0.027	2.96	0.1296
Pure error		0.038				9.133E−003		
Cor total		14.47				8.77		
*SD*	0.11				0.060			
Mean	34.71				7.48			
C.V. %	0.30				0.80			
PRESS	0.80				0.22			
*R* ^2^	0.9923				0.9959			
Adj‐*R* ^2^	0.9854				0.9922			
Pred‐*R* ^2^	0.9444				0.9744			
Adeq precision	39.885				56.123			

* Significant at p < 0.05.

The increase in the proportion of oats showed a negative impact on the OAA values in the formulation. All recipes scored higher than the acceptability limit (more than 5). A product's OAA is a highly important parameter since the sensory dimensions provide in depth insight into its marketing acceptability (Mahendradatta et al., 2020; Parn et al., 2015). Significant sum of squares and higher regression coefficients were found in all three responses to suggest conformity with the variables set with a second‐order polynomial (see Equation 2). Linear, interactive (2 FI), quadratic, and cubic models were fitted to the experimental data to achieve regression models and the results are described in Table S1. The data presented in Table S1 indicates that the quadratic model was the most suitable model for representing experimental data. The values of the coefficient of determination (*R* ^2^), the modified coefficient of determination (Adj‐*R* ^2^) and the expected coefficient of determination (Pred‐*R* ^2^) of the quadratic model were, among other models, the highest and highly significant (*p* < .001), except for the cubic model, which was aliased or confounded. The multiple regression equations (Equation 1) generated in uncoded form between the various responses and process variables are given below (Equations (4), (5)):

(4) $$\text{Firmness}=34.91+0.25\chi_{1}+0.25\chi_{2}+1.03\chi_{3}+0.13\chi_{1}\chi_{2}‐0.057\chi_{1}\chi_{3}‐0.035\chi_{2}\chi_{3}‐0.035\chi_{1}^{2}‐0.086\chi_{2}^{2}‐0.17\chi_{3}^{2}$$

(5) $$\text{OAA}=7.82+0.15\chi_{1}‐0.27\chi_{2}‐0.65\chi_{3}‐0.067\chi_{1}\chi_{2}+0.040\chi_{1}\chi_{3}‐0.18\chi_{2}\chi_{3}‐0.11\chi_{1}^{2}‐0.31\chi_{2}^{2}‐0.11\chi_{3}^{2}$$

Appropriate model parameters (*p* < .05) are *χ*_1_ (sunroot), *χ*_2_ (potato), *χ*_3_ (oats), *χ*_1_ *χ*_2_, $\chi_{2}^{2}$, $\chi_{3}^{2}$ in the case of firmness. While in case of OAA, *χ*_1_ (Sunroot), *χ*_2_ (Potato), *χ*_3_ (oats), and *χ*_1_ *χ*_2_, *χ*_2_ *χ*_3_, $\chi_{1}^{2}$, $\chi_{2}^{2}$, $\chi_{3}^{2}$ are significant model terms (*p* < .05). Regression coefficients magnitude designated maximum positive effect of oats level (1.03) followed by sunroot (0.25) and potatoes (0.25) as the same on firmness whereas increase in sunroot level affected the OAA significantly (*p* < .05).

**FIGURE 2:**
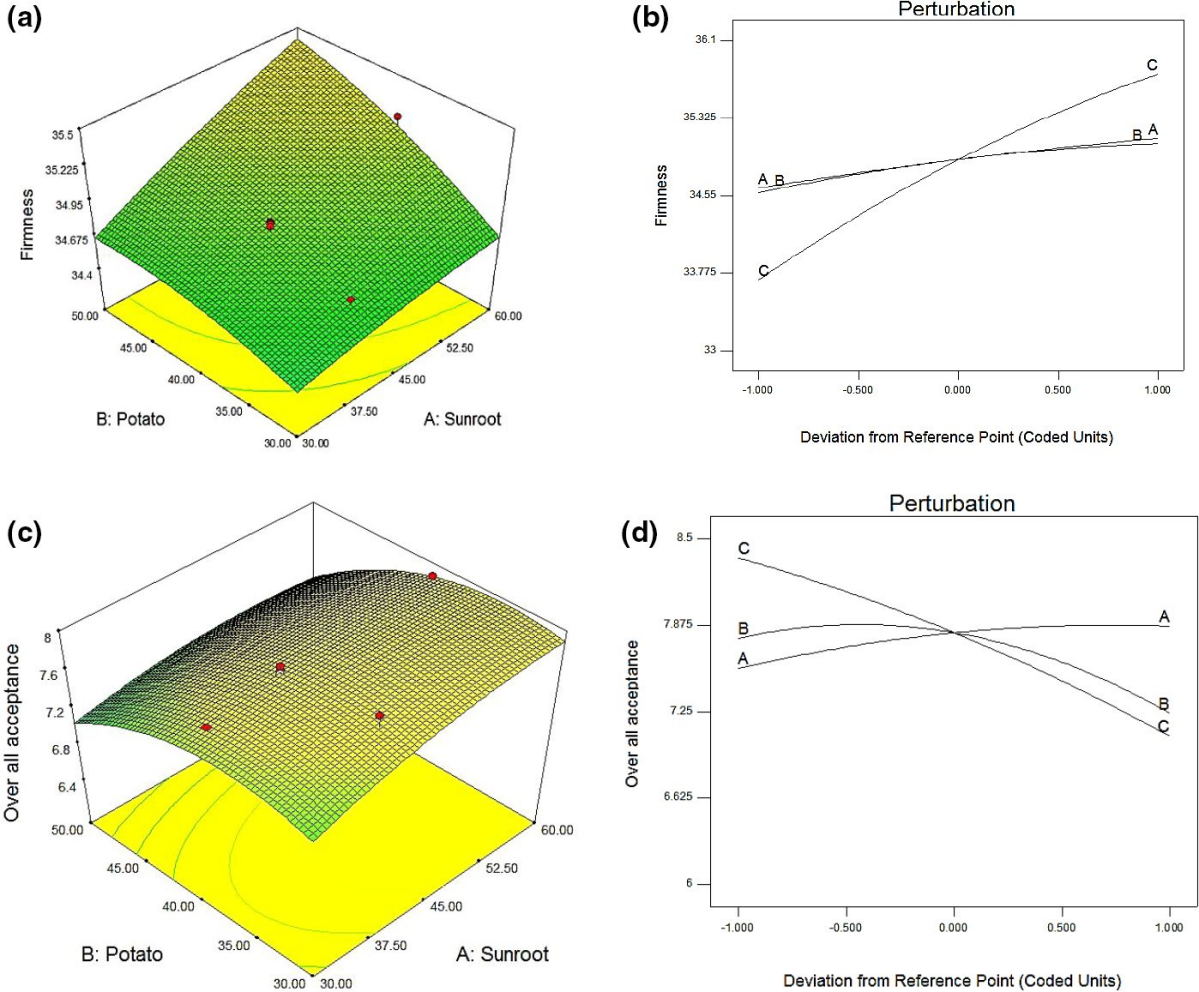
Perturbation and response surface plots for the effect of experimental variables on firmness (a and b) and overall acceptability scores (c and d) of sunroot snack bar

As illustrated in Figure 2a, b, the firmness increased with increasing sunroot (factor A), potato (factor B), and oat (factor c) concentration. On the other hand, Figure 2c, d suggested that the OAA increased with increasing sunroot (factor A), while decreased with increasing both potato (factor B) and oat (factor c) concentration. Surface plots of the model equation, disturbance, and 3D response show the significant effect of oat concentration on firmness values at higher concentration, whereas at low concentration it was less significant. The model equation, perturbation, and 3D response surface plots show the significant influence of all factors on OAA values. The sequence of the relative impact of the operating parameters on the target response was shown by the perturbation plot (Figure 2b, d) as follows for both firmness and OAA values: oats > sunroot > potato.

There were 11 solutions obtained for the optimum combination of variables with a desirability value greater than 0.981, and the optimum selected conditions were 53.99, 37.88, and 5 g, respectively, for sunroot, potato, and oat amounts. This optimal condition gave firmness and OAA (33.79 and 8.40). Sunroot snack bar was prepared in this condition again, and the experimental response values matched the predicted value using the model obtained for the optimized bar; thus, the fitted models were found suitable for predicting the responses (Table 3).

**TABLE 3:** Optimized independent variables and predicted and experimental values of responses at optimum level

Response	Predicted value	Experimental value*
Firmness (N)	33.79	33.43 ± 0.20
OAA	8.40	8.49 ± 0.18

* Data are means ± *SD* (*n* = 3).

### 3.2 Chemical, phytochemicals, and microbial contents of sunroot snack bars

Table 4 shows the chemical and phytochemicals (moisture %, ash %, fiber %, carbohydrates %, inulin %, fat %, protein %, total phenolic content, and antioxidant activity) and microbial (total plate count (TPC) and mold and yeast (M/Y)) contents of sunroot snack bars samples. The obtained results in Table 4 showed that adding flavoring to the bars did not affect the chemical, phytochemical, and microbial properties of the bar. Table 5 and Figure S2 show Phenolic profile by HPLC (mg/100 g) of fresh Sunroot and sunroot snack bar.

**TABLE 4:** Chemical, phytochemicals and microbiological profiles of fresh Sunroot and sunroot snack bars

Kind/Test	Sunroot snack bar (Control)	Sunroot snack bar with olive flavor (S1)	Sunroot snack bar with cheese flavor (S2)
Moisture %	34.8 ± 0.4 a	33.2 ± 0.3 a	32.9 ± 0.2 a
Ash %	1.30 ± 0.1 a	1.26 ± 0.2 a	1.32 ± 0.1 a
Fiber %	2.61 ± 0.05 a	2.63 ± 0.01 a	2.60 ± 0.04 a
Carbohydrates %	17.5 ± 0.3 a	17.3 ± 0.1 a	17.4 ± 0.2 a
Inulin %	5.25 ± 0.21 a	5.51 ± 0.14 a	5.43 ± 0.18 a
Fat %	0.61 ± 0.21 a	0.65 ± 0.14 a	0.63 ± 0.12 a
Protein %	2.41 ± 0.06 a	2.45 ± 0.05 a	2.43 ± 0.07 a
Phenolic content (mg/100 g)	57.08 ± 2.55 a	52.21 ± 2.32 a	55.45 ± 2.41 a
Antioxidant activity	35.25 ± 0.17 a	31.65 ± 0.11 a	33.89 ± 0.19 a
TPC (log cfu/g)	ND	ND	ND
M&Y (log cfu/g)	ND	ND	ND

Note

Different letters (a, b, c) mean statistical significant difference (*p* < .05); the results represent the mean ± standard deviation

Abbreviations: M&Y, Mold and yeast; ND, Not detected; TPC, Total plate count.

**TABLE 5:** Phenolic profile (mg/100 g) of fresh Sunroot and sunroot snack bar

Phenolic acids	Fresh sunroot	Sunroot snack bar
Gallic acid	0.87	6.37
p‐Hydroxy benzoic acid	2.93	1.32
Resvertol	22.60	8.17
Syringic acid	0.25	0.94
p‐Coumaric acid	0.50	23.57
Benzoic acid	25.13	–
Ferulic acid	0.32	–
Chlorogenic	0.82	–
Catechin	–	1.56
Pyrogallol	–	0.38
Ellagic	–	0.76

### 3.3 Consumer perception of sunroot snack bars

**FIGURE 3:**
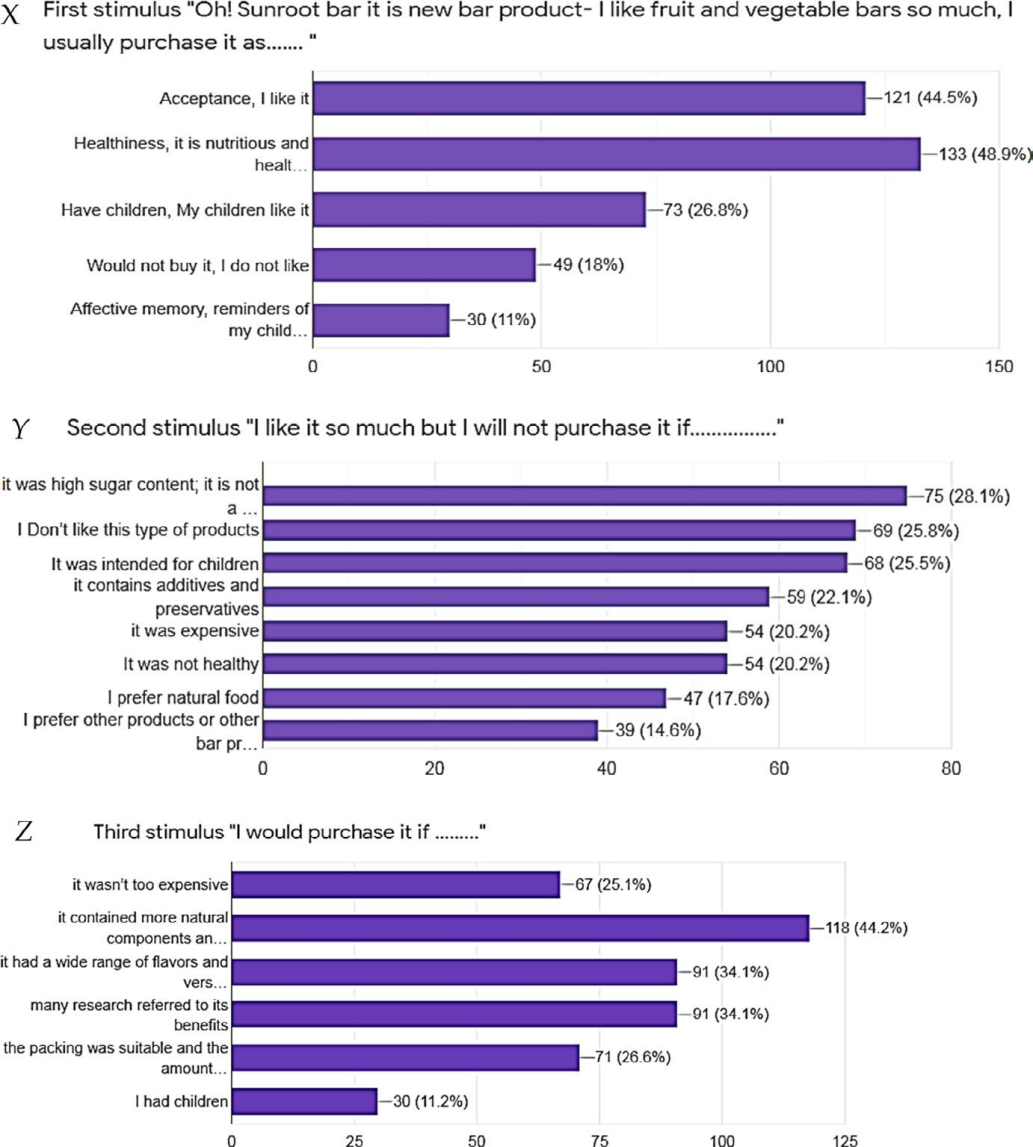
results of an online questionnaire about the three (X, Y, Z) stimuli dialogue used in the completion task

Functional foods specially products with antioxidant properties seemed to be the most promising in all world. Nowadays, Egyptian's markets have different forms of functional food products like candy, milk products, juice, bars, or cookies. Therefore, this study aimed to present a new, healthy, and low price form of fruit bar depending on the consumer knowledge and their expectations. The questionnaire was conducted in two parts as the data were presented in Figure S1 and Figure 3.

Figure S1 shows the results of the questions (part 1) for the respondents. The respondents' answers about knolwledge of sunroot plant and its nutritional value were as follows: 44% Yes, 41.4% No, and 14.7% maybe. The answers of the favorite snacks were as follows: 26% sweets, 67.5% fruits and vegtables, 35.1% bakery products, 17.7% nuts, and 0.4% few low calories cheese. Also, the responses about knowledge of the fruit or vegetable bar were as follows: 72.8% yes and 27.2% no when they asked. The answers for “How many total servings you eat of fruit or vegetable bar per day?” were as follows: 42.8% once, 37.1% twice, 12.1% third, and 6.8% Non. Did you hear about functional food? 89.1% selected yes and 10.9% selected No. The next quation was could you purchase functional food instead of commercial once? This question repononse was 51.7% yes of course, and the other were about 16%. Which consumer groups are the most consuming low calories food? 31.1% of the responses were people severe of chronic diseases (Diabetes and obesity), 26% all consumer group, 15.5% old pepole, 15.1% youth, and 11.7% children. “If sunroot bar was produced as new products, will you buy it if you know its consider a good source for minerals and phytochemical compounds, which help in lower the risk of different diseases such as intestinal problems, heart illness, cancer, and don't cause tooth decay, because most of their carbohydrate are fiber” 65.4% of the responses were yes, 33.5% maybe, and 1.1% never. The high percent of if sunroot bar meet the consumer needs was 58.6% fine followed by 28.5% well, 9.1% very well, and 3.8% badly. “How would you rate the value for money of the product (one bar 100 g for 3.05–3.13 LE)?” for this question 72.2% of the respons were accptable, 17.3% low price, and 7.5% expensive to eat daily. “Shelf life of this product is 3 months?” The rsponses were 54.8% fine, 24% well, 14.8% very well, and 6.4% badly. The response of sunroot bar nautritional value comparing with commerical types were 56.2% better, 42.3% the same, and 0.5% worse. For this quistion “If you could change just one thing about our product,” several words were written and classfied with % of each one as follows: the responses were mentioned in the following words and sentences Nothing (17.33%); Make it with different color to be suitable for children (12%); Price (9.33%); To be in different weight (2.67%); Make it with different taste or flavor (40%); and Adding some other materials such fruits, vegetables, milk, cereal, or nuts (16%). Also, Figure 3 shows the % of responses for the three (X, Y, Z) stimuli dialogue used in the completion task.

### 3.4 Storage stability of fresh Sunroot and Sunroot Snack bar samples

The presented results in Table S2 show significant changes chemicals, phytochemicals, and microbial contents of fresh sunroot. An increase in ash, fiber, TPC, and M/Y contents during storage at 4°C for 30 days was observed, with a significant decrease in moisture, carbohydrates, inulin, fat, protein, total phenolic content, and antioxidants activity. Based on these results and increase in microbial load as well as the appearance of a bitter taste, the shelf life of sunroot after 30 days was not acceptable.

**FIGURE 4:**
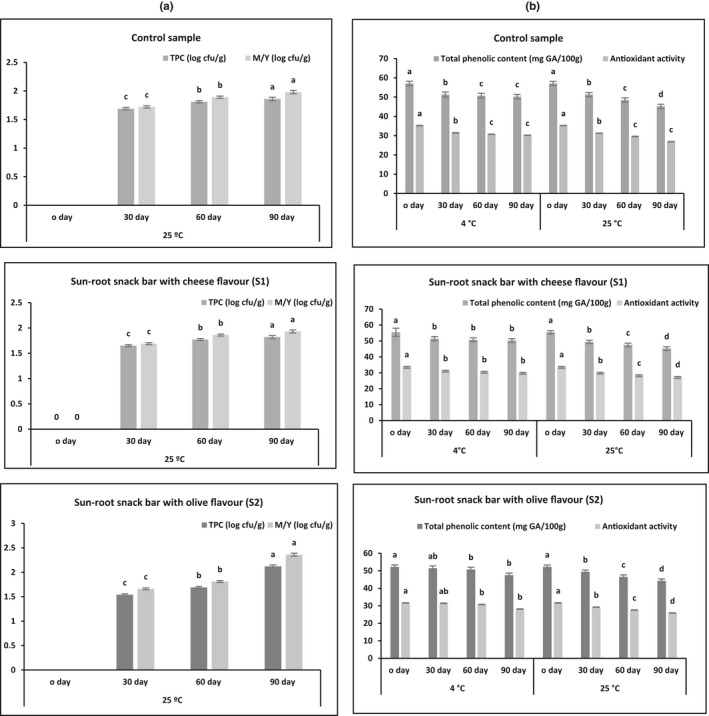
Microbial load (a) and total phenolic content and antioxidant activity (b) of sunroot snack bars (control, cheese flavor (S1) and olive flavor (S2)) during storage at 4 and 25°C for 90 days

**FIGURE 5:**
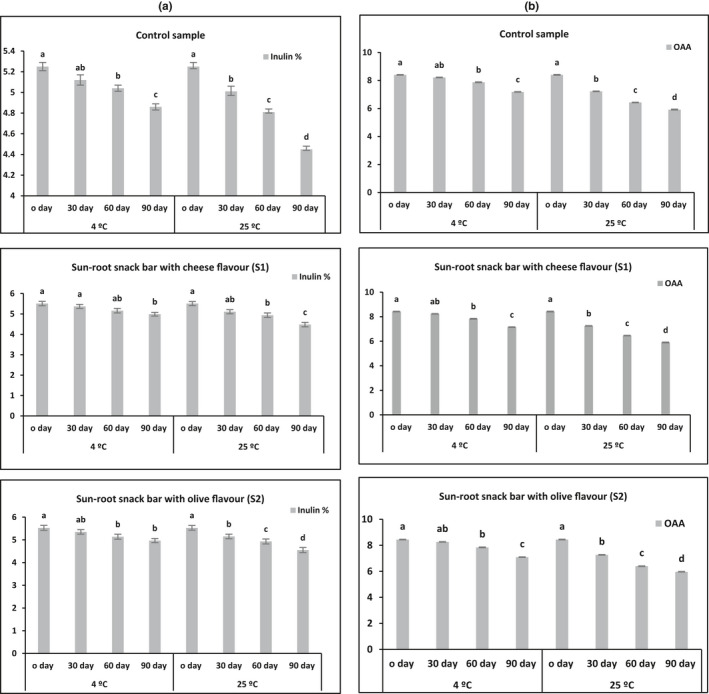
Inulin content and overall acceptability (OAA) of sunroot snack bars (control, cheese flavor (S1) and olive flavor(S2)) during storage at 4 and 25°C for 90 days

Figure 4a shows the microbial load (Total plate count, mold, and yeast) of sunroot snack bar samples during storage at 25°C for 90 days. No microbial activity was reported in all samples during storage at 4°C for 90 days was determined (data not shown, all points being zero), while a significant microbial activity was evident in sunroot bar samples during storage at 25°C for 90 days. This increase is attributed to temperature of storage. Therefore, the results indicated that the storage at 4°C was better than the storage at 25°C for more shelf life. Figure 4b shows that the total phenolic content and antioxidant activity values of sunroot snack bar samples during storage at 4 and 25°C for 90 days decreased with time. The initial total phenolic content of 52 to 57 mg/100 g, respectively, decreased to around 9 to 12% when stored at 25°C, while decreased to around 15 to 21% when stored at 4°C. There was also a decline in antioxidant activity of sunroot snack bar samples during storage at 25°C (11 to 14%) and 4°C (18 to 24%) for 90 days as compared to the initial antioxidant activity (32 to 35%). The inulin content of the sunroot bars encountered a marginal decrease of 7%–10% on storage at 4°C, and a slightly higher decrease of 15%–18% in storage at 25°C (Figure 5a). Based on the sensory results, the overall acceptability (OAA) values were decreased from 8.4–8.5 to 7.1–7.2 at 25°C, and 5.9–6.0 at 4°C (Figure 5b).

Overall, it was found that storage of sunroot snack bar samples in the refrigerator (at 4 ºC) provided a high‐quality shelf life of approximately 90 days, while at the room temperature (at 25 ºC), it was approximately one month (Figures 4 and 5). Yet, these results indicated that sunroot were microbiologically sound and sensorially acceptable for direct consumption even after the end of the indicated high‐quality shelf life.

## 4 DISCUSSION

Sunroot is a high nutritional vegetable crop, which, because of its high content of moisture, is a perishable product (which exceeds 30 days in the fridge and is not much adored in the raw form, Table S2). Developing Sunroot's ready‐to‐eat bar along with potato and oats through the optimization process could be extremely beneficial in obtaining a high‐quality food. For the optimization process, firmness and OAA were selected as responses. Firmness is a reflection of the quality of texture in any food product. In terms of cutting strength, it was evaluated to check the tenderness or toughness of the formed bar. The overall sensory acceptance values were chosen, because it depends directly on the particular composition of the product to evaluate its customer acceptability (Mahendradatta et al., 2020; Wadikar et al., 2008). From the results, a quadratic model was found to best fit the relationship, based on which optimization was conducted. Similar approaches have been used by other researchers using a CCRD design (Oberoi & Sogi, 2017; Singh et al., 2004; Yan et al., 2008). Contour plots revealed the relationship between the response and experimental levels of each (Feng et al., 2015). The *R* ^2^, adjusted‐*R* ^2^, predicted‐*R* ^2^ (should be ≥.90), and prediction error sum of squares (PRESS) were appreciably high to verify the adequacy of the model, whereas a high predicted *R* ^2^ and a low PRESS indicate a strong model fitting (Myers et al., 2016). In addition, the effects of variables were compared using the perturbation plot at a specific point in the design space. Based on the results, the optimal formulation of sunroot bar was reported.

A food product's shelf stability is assessed based on not only microbial load but also many other internal (water activity, acidity, pH, composition, usable oxygen, redox potential, and preservatives) and external (production environment, relative humidity, storage temperature, exposure to light and oxygen, and packaging content) factors (Kilcast et al., 2004). The high microbiological stability of the sunroot bars may be attributed to the osmotic pressure of the high sugar contents of the samples, which suppressed the growth of microorganisms (Pollock et al., 2017). The growth and movement of microorganisms are limited by the osmotic solution that fills plant tissue's intracellular spaces (Castello et al., 2009).

It was inferred that the sunroot bar had a high quality shelf life of 90 days at 4°C, which was shorted to a month if the bars were preserved at 25°C. However, the shelf life (of acceptable quality) at 25°C also was 90 days. The high shelf life was attributed to it being an intermediate moisture food. In intermediate moisture (IM) tomato slices, Avellaneda et al. (2012) reported that the shelf life (was 243 days) was dependent on moisture sorption and simulation isothermal models.

The data obtained from the online questionnaire were provided the essential information about consumer perceptions and knowledge about sunroot bar. However, the responses to the online questionnaire highlighted that consumers are familiar with the product (72.8% knew the fruits and vegetable bar) and 41.4% do not know what is sunroot plant. The issue related to product knowledge displays the unfamiliarity of the public with the product. On the other hand, the acceptability of sunroot bar based on the consumer perception as a new proposed in the market was very distinctive, which was introduced healthily with a suitable price of such product compared with other products in the market.

The changing phenolic and antioxidant content of sunroot snack bar samples can be attributed to polyphenols degradation, hydrophilic phenolic compounds leaching, transforming phenolics, and chemical reactions. In addition, high temperature is a major factor responsible for the reduction in phenolic contents in vegetables (Mahendradatta et al., 2020; Ismail et al., 2004). The decrease in antioxidant activity in terms of RSA was identified as a loss in total phenolic compounds (Altemimi et al., 2021; Pratap‐Singh et al., 2020). In reducing all the bioactive components, the effect of storage temperature was found significant. Castelló et al. (2011) recommended a lower osmodehydrated persimmon fruit storage temperature to maintain optimum antioxidant activity. A decline during storage in the potential of antioxidant for the IM apple product was observed (Lavelli et al., 2011). In their products, they stated that decreased water activity levels (0.55 and 0.75) maintained degradation of phytochemicals. Table 5 and Figure S2 show Phenolic profile by HPLC (mg/100 g) of fresh Sunroot and sunroot snack bar. The identification of phenolic acids in fresh sunroot tubers was mainly Benzoic acid (25.13 mg/100 g) followed by Resvertol (22.60 mg/100 g). Meanwhile, the main phenolic acids of sunroot snack bar were p‐coumaric acid (23.57 mg/100 g) followed by Resvertol (8.17 mg/100 g) and Gallic acid (6.37 mg/100 g. These differences in phenolic acid types may be due to the other additives used in processing, such as potato paste and oat powder, as well as the effects of baking temperature and time.

A food product's market acceptance mainly based on its sensory experience. Overall acceptability (OAA) values were decreased by 4 and 25°C storage for 90 days (Figure 3b). Sunroot bars stored at 4°C were found to be appropriate for up to 90 days in physicochemical and sensory characteristics. The sensory parameter that was most affected was visual acceptability, which decreased dramatically over 90 days of storage. This may be due to the browning index rising. After a storage time of 90 days, the taste of sunroot bar samples also changed negatively. The occurrence of chemically heterogeneous microenvironments leading to different physicochemical reactions may be attributed to the presence of unpleasant taste of sunroot bars upon storage (Gonzalez Viejo et al., 2020; Mahendradatta et al., 2020).

The relatively low rate of degradation of inulin (~10% at 4°C and 15% at 25°C), greater shelf‐life of sunroot bar as compared to fresh sunroot, and its high acceptability in sensory trials suggested the formulation of an inulin‐rich healthy snack bar. Human intestinal enzymes cannot digest the inulin with β‐2,1 linkages between fructose monomers, resulting in important applications in functional foods appropriate for the management of Diabetes, obesity, and other health conditions associated with blood sugar (Yang et al., 2015). Moreover, Franck (2002) reported that, the nondigestible nature of inulin contributes in a caloric value that is lower than that of other traditional carbohydrates, as energy is only obtained from the fermentation metabolism of fatty acids and lactate. Inulin can also be used to substitute fat, sugar and flour in milk products, cereals, and baked goods for calorie reduction purposes. Therefore, Stamataki et al. (2016) tried to increase the dietary fiber content and investigate additional potential postprandial benefits, enrichment biscuits snacks with inulin, a fructooligosaccharides (FOS) acting as soluble fiber. In addition, they demonstrated the biscuits with Oat flicks and inulin scored higher for all the evaluated sensory properties, besides texture and achieved higher overall acceptance scores.

Moreover, in some cases, the prices of the materials used in the production process can also have an impact on the price of the fruits and vegetables snack bar, which the recent high price of dried fruits for example dried mango (20–27 €/kg), dried pineapples (15–20 €/kg), and dried banana (6–10 €/kg) led to increasing the price in the international side. Therefore, the possibility of transfer this price to be lower with maintaining added value in the form of sustainable, natural, or acceptable nutritional value products. For this reason, the sunroot bar as one of the new proposed snack bar products is estimated to represent a good reduction in the price to more than 90% of the world retail price and 70% of in the national market of commercial fruit and vegetable bar. The price of a sunroot bar “100 g” was calculated to be 3.09–3.13 LE its equal 0.17 €, while for the world retail price of commercial fruit and vegetable bar was 3 € per 100 g and the same product in the national market equal 13.40 LE its equal 0.72 per 100 g.

In order to make the pricing decision, which reflect the quality of food and which also based on the shelf life for processed food. Therefore, the analyzing of relationship between the calculated price (0.17 €), the fluctuation of quality level, and the change in shelf life of sunroot bar, which reach 3 months with acceptable microbiological levels for human consumption. Finally, depending on the bundling selling based on shelf life can bring higher profits to retailers and make the sunroot bar available for all consumer groups.

## 5 CONCLUSIONS

Based on the results from this study, it could be concluded that sunroot could be used directly as a replacement for conventional white flour snacks during formulation of snack bars. Higher sunroot content was found to increase both firmness and overall acceptability of the bar. A quadratic model was found to best describe the relationship of firmness and overall sensory acceptability with product composition. During shelf‐life tests, the storage temperature was found to influence the shelf stability of the bar. The developed sunroot bar was rich in inulin, phenolic, and antioxidants and can thus be considered a functional food for consumers seeking vegan allergen‐free bars.

## CONFLICTS OF INTEREST

The authors declare no conflict of interest.

## AUTHOR CONTRIBUTIONS

**Tarek Gamal Abedelmaksoud:** Conceptualization (equal); Data curation (equal); Formal analysis (equal); Methodology (equal); Project administration (equal); Validation (equal); Visualization (equal); Writing‐original draft (equal). **Sayed Saad Smuda:** Formal analysis (equal); Methodology (equal); Visualization (equal); Writing‐original draft (equal). **Ammar B. Altemimi:** Conceptualization (equal); Investigation (equal); Project administration (equal); Writing‐original draft (equal); Writing‐review & editing (equal). **Reda Mahgoub Mohamed:** Methodology (equal); Supervision (equal); Visualization (equal); Writing‐review & editing (equal). **Anubhav Pratap‐Singh:** Conceptualization (equal); Funding acquisition (equal); Investigation (equal); Project administration (equal); Supervision (equal); Visualization (equal); Writing‐original draft (equal); Writing‐review & editing (equal). **Marwa Rashad Ali:** Conceptualization (equal); Project administration (equal); Supervision (equal); Writing‐review & editing (equal).

## Supporting information

Supplementary Material
