# Supplementary material for: Sunroot snack bar: Optimization, characterization, consumer perception, and storage stability assessment
Source: Food Sci Nutr. 2021 Jun 29;9(8):4394–407. doi: 10.1002/fsn3.2412 (PMC8358387; doi:10.1002/fsn3.2412)
Supplement: Supplementary file 1 — Supplementary Material [file FSN3-9-4394-s001.docx]

**Supplementary Materials:** Table S1: Adequacy of the model tested, Table S2: Chemical, phytochemicals and microbiological profiles of fresh Sun-root and sun-root snack bars, Table S3 trials 1 and 2 for optimizing concentration (g) of sun-root, potato (g) and Oats (g)added to produce sun-root snack bar; Table S4 Sensory mean scores of trials 1and 2 ; Figure S1 results of part 1 questions that concentrated on the objective which was to see the consumer perception degree about fruits and vegetables bars in general and sunroot bar in particularly.

**Table S1. Adequacy of the model tested**

| **Source** | **Std. Dev.** | **R^2^** | **Adjusted R^2^** | **Predicted R^2^** | **PRESS** | **p-value**  **Prob > F** | **Remarks** |
| --- | --- | --- | --- | --- | --- | --- | --- |
| **Firmness (N)** | | | | | | | |
| **Linear** | 0.20 | 0.9554 | 0.9471 | 0.9249 | 1.09 | 0.0205 |  |
| **2FI** | 0.20 | 0.9623 | 0.9450 | 0.8461 | 2.23 | 0.0163 |  |
| **Quadratic** | 0.11 | 0.9923 | 0.9854 | 0.9444 | 0.80 | 0.2476 | Suggested  Aliased |
| **Cubic** | 0.088 | 0.9974 | 0.9899 |  |  | + |  |
| **OAA** | | | | | | | |
| **Linear** | 0.30 | 0.8338 | 0.8026 | 0.7064 | 2.57 | < 0.0001 |  |
| **2FI** | 0.30 | 0.8664 | 0.8048 | 0.3587 | 5.62 | < 0.0001 |  |
| **Quadratic** | 0.060 | 0.9959 | 0.9922 | 0.9744 | 0.22 | 0.1296 | Suggested  Aliased |
| **Cubic** | 0.043 | 0.9990 | 0.9960 |  |  | + |  |

Quadratic models are underlined and the model is labeled as “Suggested” by the Design-Expert software. Suggestion is based on a subjective

scoring system that uses a combination of selected metrics to propose that quadratic model looks best compared with other models

**Table S2. Effect of storage time (for 30 days at 4 ºC) on chemical, phytochemicals and microbiological contents of fresh Sun-root**

| **Kind / Test** | **Storage time per day** | | | |
| --- | --- | --- | --- | --- |
|  | **Zero** | **10** | **20** | **30** |
| **Moisture%** | 81.12±0.41a | 80.05±0.32b | 79.85±0.17c | 78.46±0.20d |
| **Ash%** | 0.81±0.03b | 0.83±0.02ab | 0.85±0.07a | 0.88±0.05a |
| **Fiber%** | 3.24±0.12a | 3.27±0.15a | 3.28±0.11a | 3.31±0.18a |
| **Carbohydrates %** | 15.76±0.23a | 15.36±0.27a | 14.46±0.28b | 13.87±0.16c |
| **Inulin %** | 11.34±0.32a | 11.14±0.22a | 10.36±0.26b | 9.71±0.21c |
| **Fat%** | 0.22±0.15a | 0.21±0.11a | 0.19±0.15b | 0.14±0.17c |
| **Protein%** | 2.10±0.17a | 2.04±0.16a | 1.98±0.13b | 1.83±0.18c |
| **Total phenolic content (mg GA/100g)** | 75.22±2.55a | 72.11 ± 2.11b | 71.22 ± 1.77c | 69.22 ± 2.21d |
| **Antioxidant activity** | 41.22± 0.15a | 39.36± 0.12b | 38.87± 0.12c | 36.56± 0.10d |
| **TPC (log cfu/g)** | 1.65±0.11d | 1.85±0.12c | 2.55±0.11b | 3.25±0.15a |
| **M/Y (log cfu/g)** | 1.69±0.10d | 1.95±0.12c | 2.87±0.13b | 3.75±0.12a |

*ND: means not detected; Different letters (a, b, c) mean statistical significant difference (p<0.05); the results represent the mean ± standard deviation

**Table S3 trials 1 and 2 for optimizing concentration (g) of sun-root, potato (g) and Oats (g)added to produce sun-root snack bar**

| Trial 1 for adjusting concentration (g) of sun-root with potato added to produce sun-root snack bar | | | | | | |
| --- | --- | --- | --- | --- | --- | --- |
| Materials | **Sun-Root (g)** | **Creamy cheese (g)** | **Potato (g)** | **Peanuts (g)** | **Oats (g)** | **Olive or Cheese flavour (g)** |
| Trial 1 Conc. 1 | 10 | 13 | 65 | 8 | 5 | 3-5 |
| Trial 1 Conc. 2 | 20 | 13 | 65 | 8 | 5 | 3-5 |
| Trial 1 Conc. 3 | 30 | 13 | 55 | 8 | 5 | 3-5 |
| Trial 1 Conc. 4 | 35 | 13 | 50 | 8 | 5 | 3-5 |
| Trial 1 Conc. 5 | 40 | 13 | 45 | 8 | 5 | 3-5 |
| Trial 1 Conc. 6 | 45 | 13 | 40 | 8 | 5 | 3-5 |
| Trial 1 Conc. 7 | 50 | 13 | 35 | 8 | 5 | 3-5 |
| Trial 1 Conc. 8 | 55 | 13 | 30 | 8 | 5 | 3-5 |
| Trial 1 Conc. 9 | 60 | 13 | 20 | 8 | 5 | 3-5 |
| Trial 1 Conc. 10 | 65 | 13 | 10 | 8 | 5 | 3-5 |
| Trial 2 for adjusting Oats concentration (g) (either olive flavour or cheese flavour) added to sun-root snack bar | | | | | | |
| Materials | **Sun-root (g)** | **Creamy cheese (g)** | **Potato (g)** | **Peanuts (g)** | **Oats (g)** | **Olive or Cheese flavor (g)** |
| Trial 2 Conc. 1 | 45 | 13 | 40 | 8 | 5 | 3-5 |
| Trial 2 Conc. 2 | 45 | 13 | 40 | 8 | 10 | 3-5 |
| Trial 2 Conc. 3 | 45 | 13 | 40 | 8 | 15 | 3-5 |
| Trial 2 Conc. 4 | 45 | 13 | 40 | 8 | 20 | 3-5 |
| Trial 2 Conc. 5 | 45 | 13 | 40 | 8 | 25 | 3-5 |
| Trial 2 Conc. 6 | 45 | 13 | 40 | 8 | 30 | 3-5 |

***Cheese flavour also with the same concertation of olive flavour**

**Table S4 Sensory mean scores of trials 1and 2**

| Kind / Feature | Colour | Odour | Taste | Texture | Over all acceptance |
| --- | --- | --- | --- | --- | --- |
| Trial 1 | | | | | |
| Olive flavor Conc. 1 | 6.40±0. 3c | 6.81±0.2d | 6.70±0.2c | 6.65±0.3c | 6.61±0.3c |
| Olive flavor Conc. 2 | 6.57±0.2c | 7.84±0. 2b | 7.40±0.2b | 6.90±0.2c | 6.92±0.4c |
| Olive flavor Conc. 3 | 7.51±0.1a | 7.70±0. 2b | 7.20±0.2b | 7.60±0.2b | 7.70±0.3b |
| Olive flavor Conc. 4 | 8.57±0.3a | 8.62±0.5a | 8.10±0.3a | 8.51±0.3a | 8.53±0.2a |
| Olive flavor Conc. 5 | 8.50±0.2a | 8.67±0. 2a | 8.20±0.2a | 8.30±0.2a | 8.46±0.4a |
| Olive flavor Conc. 6 | 8.44±0.3a | 8.54±0.1a | 8.41±0.4a | 8.33±0.2a | 8.47±0.3a |
| Olive flavor Conc. 7 | 8.40±0.2a | 8.36±0.6a | 8.32±0.5a | 8.31±0.3a | 8.39±0.4a |
| Olive flavor Conc. 8 | 8.45±0.5a | 8.11±0.5a | 8.23±0.4a | 8.23±0.2a | 8.55±0.3a |
| Olive flavor Conc. 9 | 7.41±0.3b | 7.59±0.1c | 7.56±0.4b | 7.33±0.2b | 7.40±0.3b |
| Olive flavor Conc. 10 | 7.28±0.2b | 7.32±0.6c | 6.41±0.5c | 6.42±0.3c | 7.15±0.4b |
| Cheese flavor Conc. 1 | 6.70±0.1c | 6.41±0.3c | 6.50±0.2c | 6.90±0.6 | 6.70±0.2 |
| Cheese flavor Conc. 2 | 6.53±0.1c | 6.79±0.3c | 6.63±0.2c | 6.54±0.6c | 6.61±0.3c |
| Cheese flavor Conc. 3 | 7.42±0.2b | 7.73±0.3b | 7.47±0.2b | 7.82±0.3b | 7.68±0.4b |
| Cheese flavor Conc. 4 | 8.32±0.1a | 8.40±0.3a | 8.30±0.2a | 8.70±0.6a | 8.40±0.2a |
| Cheese flavor Conc. 5 | 8.20±0.4a | 8.71±0. 2a | 8.25±0.3a | 8.13±0.3a | 8.38±0.5a |
| Cheese flavor Conc. 6 | 8.34±0.3ba | 8.54±0.1a | 8.60±0.4a | 8.33±0.2a | 8.47±0.3a |
| Cheese flavor Conc. 7 | 8.27±0.2ba | 8.30±0.6a | 8.32±0.5a | 8.30±0.3a | 8.22±0.4a |
| Cheese flavor Conc. 8 | 8.45±0.5ca | 8.11±0.5a | 8.23±0.4a | 8.23±0.2a | 8.55±0.3a |
| Cheese flavor Conc. 9 | 7.33±0.3b | 7.57±0.1b | 7.61±0.4b | 7.39±0.2b | 7.45±0.3b |
| Cheese flavor Conc. 10 | 7.27±0.2b | 7.39±0.6b | 7.36±0.5b | 7.34±0.3b | 7.22±0.4b |
| Trial 2 | | | | | |
| Olive (Conc.1) | 8.50±0.4a | 8.10±0.2a | 8.35±0.6a | 8.39±0.2a | 8.56±0.3a |
| Olive (Conc.2) | 8.30±0.3a | 8.50±0.3a | 8.20±0. 2a | 8.10±0.2a | 8.62±0.2a |
| Olive (Conc.3) | 8.70±0.4a | 8.40±0.4a | 8.50±0.5a | 7.40±0.5b | 8.70±0.3a |
| Olive (Conc.4) | 7.87±0.3b | 7.64±0.2b | 7.84±0.3b | 6.60±0.5c | 7.93±0.4b |
| Olive (Conc.5) | 5.21±0.4c | 5.86±0.3c | 5.43±0.5c | 5.20±0.5d | 5.35±0.3c |
| Olive (Conc.6) | 5.21±0.4c | 5.84±0.3c | 5.40±0.5c | 5.10±0.5d | 5.30±0.2c |
| Cheese (Conc.1) | 8.30±0.3a | 8.19±0.4a | 8.35±0.2a | 8.38±0.4a | 8.57±0.2a |
| Cheese (Conc.2) | 8.40±0.2a | 8.15±0.3a | 8.40±0.3a | 8.05±0.3a | 8.45±0. 4a |
| Cheese (Conc.3) | 8.34±0.3a | 8.04±0.1a | 8.30±0.4a | 7.33±0.2b | 7.97±0.3b |
| Cheese (Conc.4) | 7.20±0.2b | 7.30±0.6b | 7.32±0.5b | 6.30±0.3c | 7.12±0.4c |
| Cheese (Conc.5) | 6.45±0.5c | 6.11±0.5c | 5.23±0.4b | 5.23±0.2d | 5.55±0.3d |
| Cheese (Conc.6) | 5.23±0.4d | 5.81±0.3d | 7.42±0.5c | 5.20±0.5d | 5.36±0.4d |

***Different letters (a, b, c) mean statistical significant difference (p<0.05); the results represent the mean ± standard deviation**

| 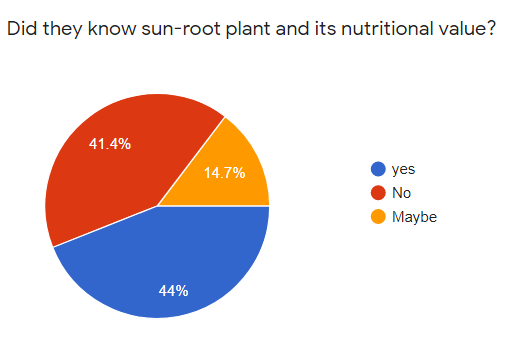 | 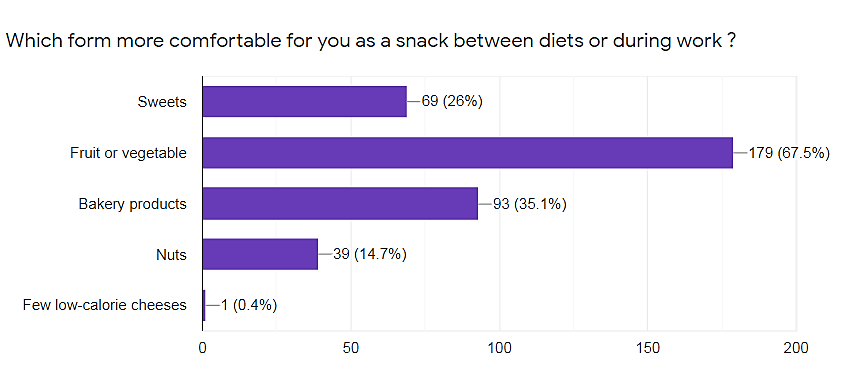 |
| --- | --- |
| 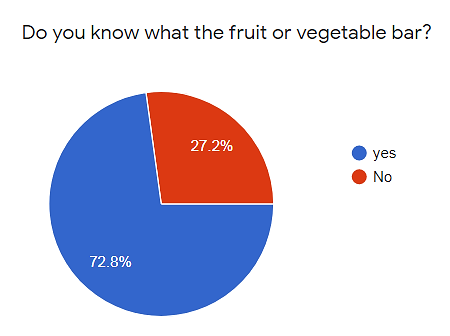 | 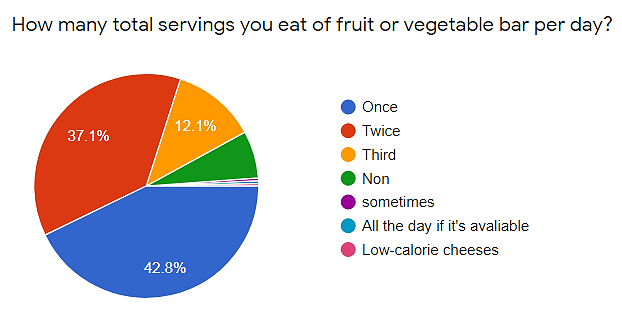 |
| 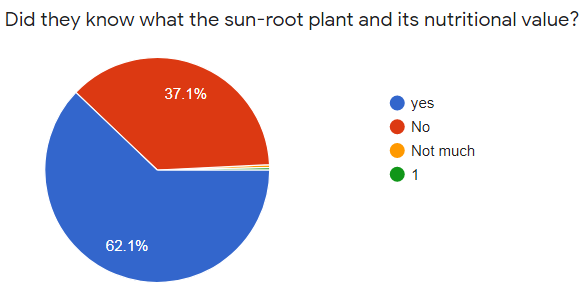 | 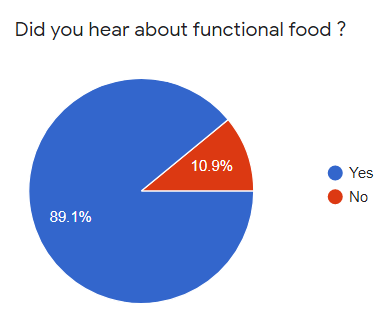 |
| 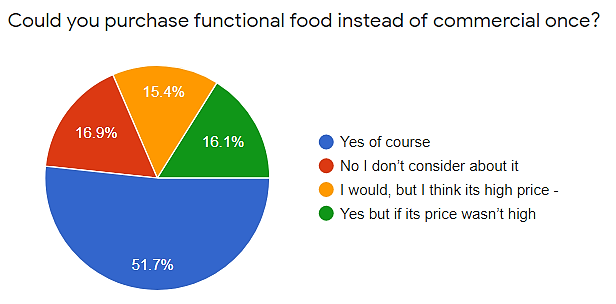 | 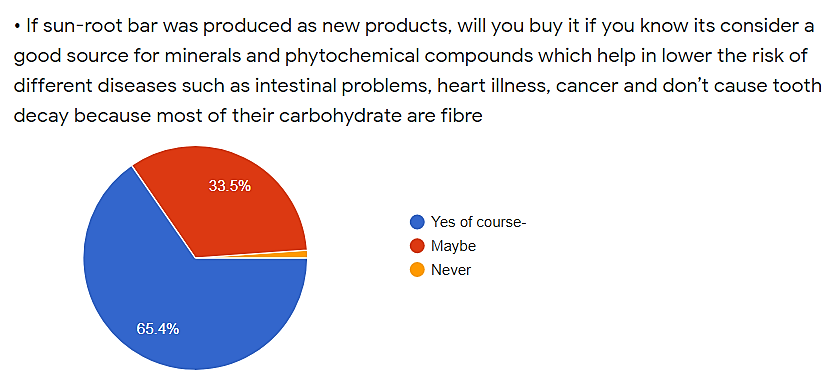 |
| 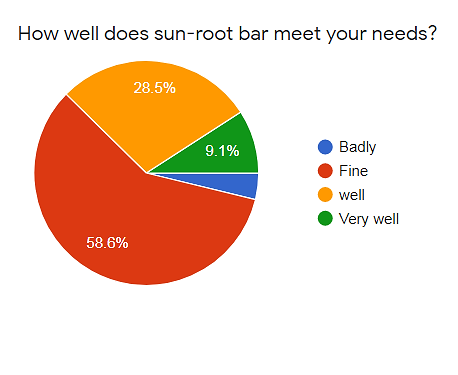 | 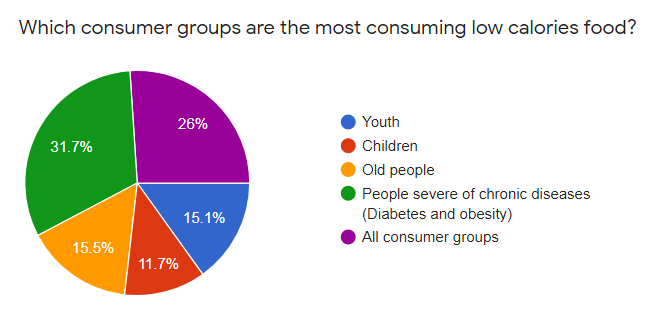 |
| 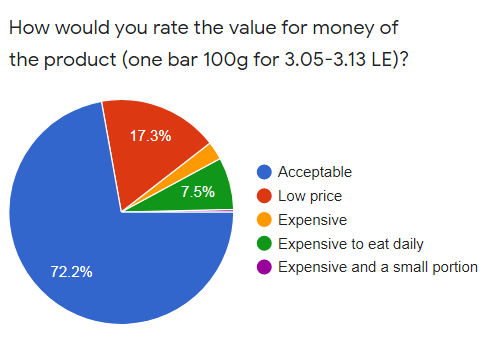 | 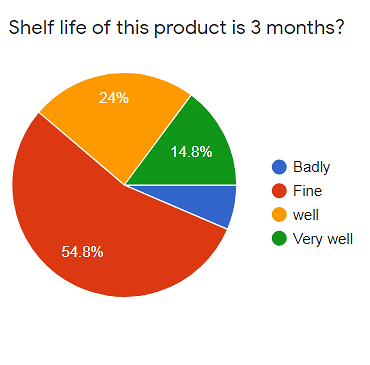 |
| 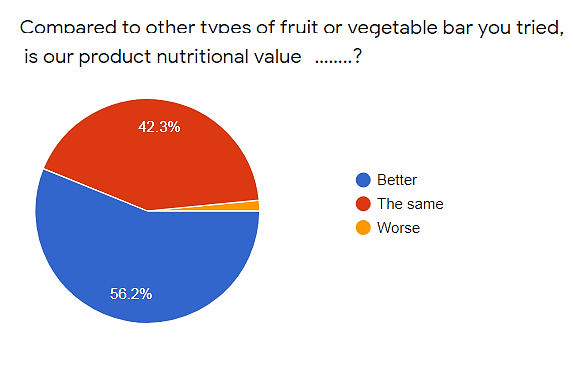 | |

**Figure S1 results of Part 1 questions that concentrated on the objective which was to see the consumer perception degree about fruits and vegetables bars in general and sunroot bar in particularly.**

| 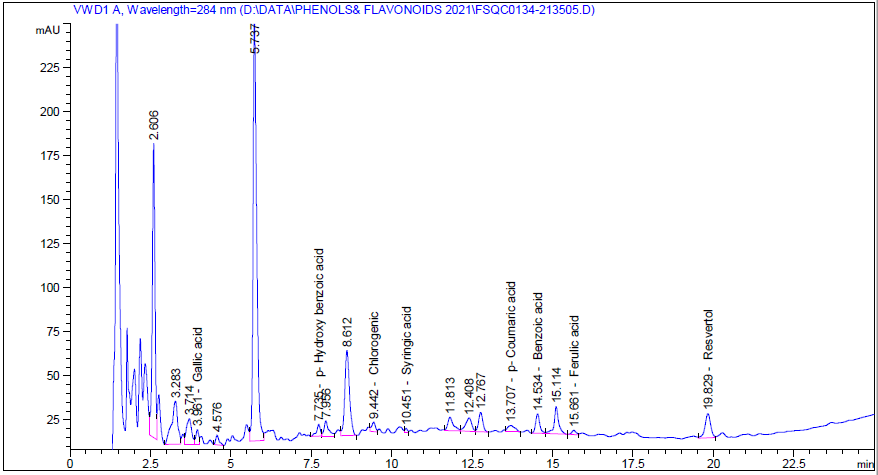 **(A)** |
| --- |
| 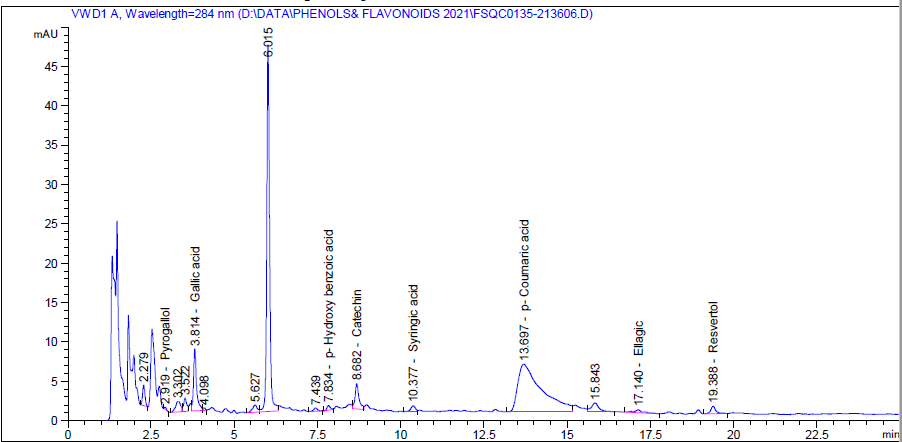 **(B)** |

**Figure S2 chromatograms of phenolic compounds for fresh sunroot (A) and sun-root snack bar (B)**
